# SUBJECTIVE COGNITIVE DECLINE IN OLDER ADULTS BY SEXUAL ORIENTATION AND GENDER IDENTITY

**DOI:** 10.1093/geroni/igae098.0167

**Published:** 2024-12-31

**Authors:** Ellesse-Roselee Akre, Katherine Miller, Harry Barbee, Tara McKay

**Affiliations:** Johns Hopkins Bloomberg School of Public Health, Baltimore, Maryland, United States; Johns Hopkins University, Baltimore, Maryland, United States; Johns Hopkins Bloomberg School of Public Health, Baltimore, Maryland, United States; Vanderbilt University, Nashville, Tennessee, United States

## Abstract

LGBTQ+ adults experience a high risk of subjective cognitive decline (SCD) due to increased exposure to stressors that threaten cognitive health. Yet, researchers know little about how the risk of SCD varies by sexual orientation and gender identity (SOGI). We examine how SCD differs by SOGI using Wave 1 of the Vanderbilt University Social Networks, Aging, and Policy Study survey of LGBTQ+ adults aged 50-76 residing in the South (n=1256). We assess subjective cognitive health using an index of self-reported cognitive difficulties, many of which have been used in validated instruments. Using linear probability models, we examine the association of SCD with SOGI. To identify significant differences, we use standardized differences >10% in the average predicted probability of reporting ≥1 cognitive difficulty across SOGI, after adjusting for sociodemographic characteristics. Forty percent of respondents reported ≥1 cognitive difficulty. Bisexual individuals have the highest likelihood of SCD (54%), while lesbian/gay respondents had the lowest (39%). Across gender identities, gender non-conforming persons have the highest predicted probability of SCD (57%), followed by transgender men (54%), transgender women (48%), females/assigned female at birth (40%), and males/assigned male at birth (39%). Standardized differences are >10% across all comparisons. We find evidence that the prevalence of ≥1 cognitive difficulty in the sample of LGBTQ+ adults varies significantly by SOGI. Comprehensive SOGI health data from all US states is essential to accurately gauge SCD prevalence among LGBTQ+ older adults and to ensure inclusive clinical and public health interventions for populations at higher risk of cognitive impairment.

